# Postmortem computed tomography assessment of skeletal and dental age in Polish children, adolescents, and young adults

**DOI:** 10.1007/s12024-023-00662-x

**Published:** 2023-07-10

**Authors:** Oleksiy Lopatin, Marta Barszcz, Anna Jurczak, Krzysztof Jerzy Woźniak

**Affiliations:** 1 Chair and Department of Forensic Medicine, Faculty of Medicine, Jagiellonian University Medical College, Grzegorzecka 16, 31-531 Krakow, Poland; 2 Doctoral School of Medical and Health Sciences, Jagiellonian University Medical College, Krakow, Poland; 3 Department of Environmental Health, Institute of Public Health, Faculty of Health Science, Jagiellonian University Medical College, Krakow, Poland

**Keywords:** Postmortem computed tomography, Skeletal age estimation, Dental age estimation, Secondary ossification centers, Permanent teeth, Age estimation

## Abstract

This paper presents a retrospective analysis of postmortem computed tomography (PMCT) scans of secondary ossification centers in the medial clavicular epiphysis, iliac crest apophysis, proximal humeral epiphysis, distal femoral epiphysis, proximal tibial epiphysis, and distal tibial epiphysis. At the same time, we analyzed PMCT scans of the maxillary and mandibular incisors, canines, premolars, and molars. We assessed 203 corpses, whose age ranged from 2 to 30 years, including 156 males and 47 females. The purpose of our study was to compare the processes of secondary ossification center fusion and permanent tooth maturation. Our research hypothesis was that certain stages of skeletal and dental maturation occur along consistent timelines that can be related to the chronological age. Secondary ossification center fusion was evaluated based on Kreitner and also McKern and Steward’s classifications. The process of permanent tooth maturation was evaluated with Demirjian’s method. Spearman’s correlation coefficients (Rho) were positive in all analyses, which indicates that epiphyseal fusion progresses with age. The strongest relationship between the age and the stages of ossification was observed in the proximal tibial epiphysis (*p* < 0.001; Rho = 0.93) in females and in the medial clavicular epiphysis (*p* < 0.001; Rho = 0.77) in males. Studies show the importance of concomitant analysis of skeletal and dental maturation with a subsequent comparison of the results to achieve a greater precision in age estimation. A comparison of the results obtained in the study population of Polish children, adolescents, and young adults with the results of other studies in populations of similar ages showed a number of similarities in the time windows of dental and skeletal maturation. These similarities may help in age estimation.

## Introduction

From the beginning of the twenty-first century, there has been an increase in the number of radiological assessments in children, adolescents, and young adults. Most of these assessments are conducted in the living. Bone age assessments in children, adolescents, and young adults are based on the presence or absence of primary and secondary ossification centers and epiphyseal fusion [1, 2]. Various radiographic methods are also used for dental age estimation. The ossification centers most commonly used for age assessment are those in the medial clavicular epiphysis and the ossification centers of the hand. These methods, as well as dental age assessment, have been recommended by the Study Group on Forensic Age Diagnostics [3]. Over the last two decades, magnetic resonance imaging (MRI) has become the most popular method of radiological age assessment in the living. This popularity of MRI is due to the lack of harmful electromagnetic radiation. Conversely, postmortem age assessments conducted at forensic medicine institutes are based chiefly on X-ray imaging, including computed tomography (CT). Postmortem use of MRI is limited due to the high cost of this technique [4]. The first postmortem computed tomography (PMCT) examinations in Poland were conducted by the Forensic Medicine Department and the Radiology Department of the Jagiellonian University Medical College in 2009 [5]. Since 2012, the Forensic Medicine Department has its own CT equipment. Since then, the cumulative number of examined corpses has been gradually increasing, with some of the collected data used for the present study.

The purpose of this study was to estimate the age in Polish children, adolescents, and young adults with the use of PMCT by analyzing the phases of secondary ossification center fusion and to estimate dental age by analyzing features of dental maturation in permanent dentition. Subsequently, we compared the results of various relevant studies in this field, and compared the estimated age of individuals at each stage of secondary center ossification with their chronological age, with the subadult–adult cutoff adopted at the legal age of majority, i.e., 18 years, in order to evaluate the usefulness of these assessments in legal cases. The results of such studies may be helpful in estimating the age of minors. Another purpose of our study was to compare the fusion process at multiple secondary ossification centers against the process of permanent tooth maturation. Other authors have typically evaluated bone maturation based on a single ossification center. Our study shows the advantage of using PMCT on cadavers rather than X-rays or CT scans on live individuals because clinical radiology modalities are not recommended in individuals.

## Material and methods

This retrospective analysis of CT scans visualizing secondary ossification centers at the medial clavicular epiphysis, iliac crest apophysis, proximal humeral epiphysis, distal femoral epiphysis, proximal tibial epiphysis, and distal tibial epiphysis. At the same time, CT scans of the maxillary and mandibular incisors, canines, premolars, and molars were analyzed. The PMCT examinations had been conducted at the Forensic Medicine Department of the Jagiellonian University Medical College in the years 2012–2016. The equipment used was a Siemens Somatom Emotion 16 computed tomography scanner, 130 kV, 240 mAs, collimation 16 × 0.6, pitch 0.85, and slice thickness of 0.75 mm for the head and of 1.5 mm for the torso and limbs. Image reconstruction was obtained via OsiriX software v.5.5.1, Pixmeo SARL, Switzerland.

All PMCT examinations had been conducted at the Forensic Medicine Department, with a Siemens Somatom Emotion 16 computed tomography scanner, since a conventional orthopantomography (OPG) equipment was not available. Nonetheless, Brough et al. [6] demonstrated non-inferiority of CT scans compared with conventional radiographs in estimating dental age. Therefore, we decided that dental age assessments may be complemented by using CT scans.

Statistical analyses were conducted with IBM SPSS Statistics 28 software.

Spearman’s Rho test was used to check if linear connection between changes in ossification center and age occurs. The level of statistical significance was adopted at *p* = 0.05. Descriptive statistics were used to analyze dentition-related data.

The Wilcoxon signed-rank test was used to assess whether the methods of assessing the changes in ossification centers and tooth maturity were justifiable.

**Fig. 1:**
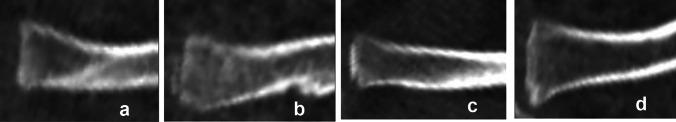
Computed tomography scans showing the process of ossification and subsequent fusion at the medial clavicular epiphysis based on Kreitner’s four-stage classification [7]. **a** Stage 1—epiphysis not ossified. **b** Stage 2—epiphysis ossified without any fusion. **c** Stage 3—partial epiphyseal fusion. **d** Stage 4—complete epiphyseal fusion; image source: in-house archives

**Fig. 2:**
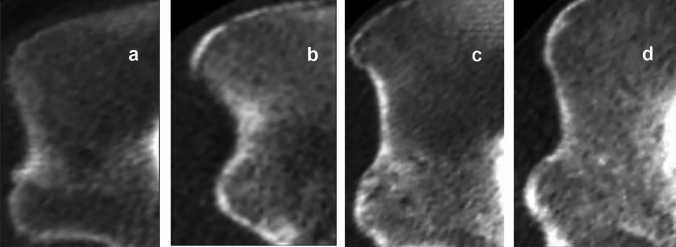
Computed tomography images depicting the stages of ossification and fusion of the iliac crest apophysis based on Kreitner’s four-stage classification [8]. **a** Stage 1—no ossification of the iliac crest apophysis. **b** Stage 2—iliac crest apophyseal ossification without any fusion with the ilium. **c** Stage 3—partial fusion. **d** Stage 4—complete fusion; image source: in-house archives

**Fig. 3:**
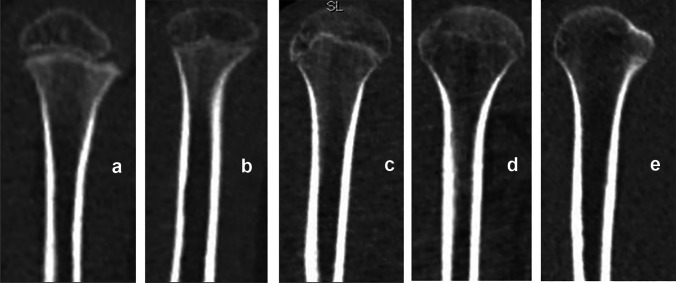
The process of proximal humeral epiphyseal fusion, based on McKern and Steward’s classification [10]. **a** Stage 0—non-union (no epiphyseal fusion, with a complete radiolucent gap visible between the epiphysis and diaphysis). **b** Stage 1—beginning of union (the epiphyseal gap is no longer complete, but more than half of its length remains radiolucent). **c** Stage 2—active union (the terminal plate of the epiphysis can no longer be distinguished and less than half of the epiphyseal gap remains radiolucent). **d** Stage 3—recent union (the epiphysis and diaphysis are completely fused forming a single bone, with a metaphyseal line possibly remaining at the border between the epiphysis and diaphysis). **e** Stage 4—complete union (all traces of epiphyseal differentiation have disappeared); image source: in-house archives

**Fig. 4:**
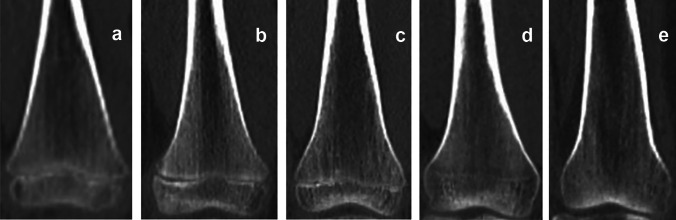
The process of distal femoral epiphyseal fusion, based on McKern and Steward’s classification [10]: **a**–**e** Stages 0–4, respectively; image source: in-house archives

**Fig. 5:**
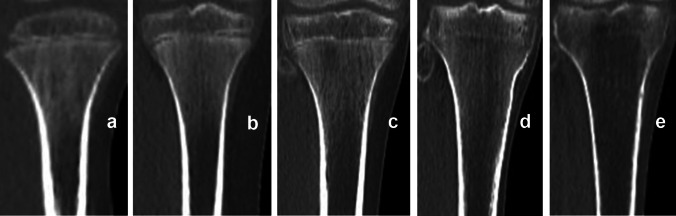
The process of proximal tibial epiphyseal fusion, based on McKern and Steward’s classification [10]: **a**–**e** Stages 0–4, respectively; image source: in-house archives

**Fig. 6:**
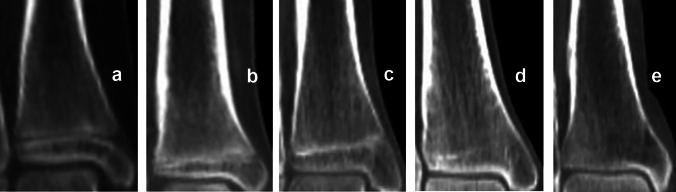
The process of distal tibial epiphyseal fusion, based on McKern and Steward’s classification [10]: **a**–**e** Stages 0–4, respectively; image source: in-house archives

**Fig. 7:**
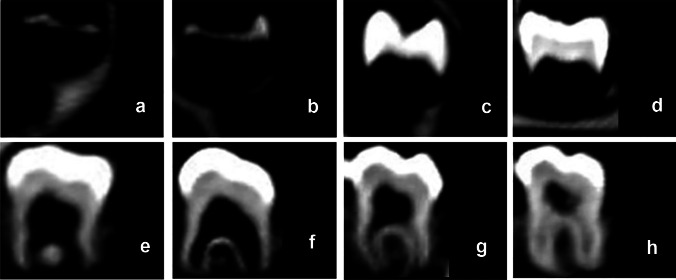
Computed tomography images depicting the process of permanent tooth maturation, classified based on Demirjian’s method [2, 12]. **a** Stage A—beginning of calcification (no fusion of single calcified occlusal points). **b** Stage B—calcification points fuse, the contour of the occlusal surface is recognizable. **c** Stage C—enamel formation has been completed, dentin formation has begun. **d** Stage D—crown formation is complete. **e** Stage E—root formation, the radicular bifurcation forms in molars. **f** Stage F—the root length is equal to or greater than the crown height. **g** Stage G—the roots have almost reached their full length, the apical end is partially open. **h** Stage H—the root apex is completely closed; image source: in-house archives

Images of 203 corpses of individuals (156 males and 47 females) aged 2–30 years were evaluated. Secondary ossification centers in the medial clavicular epiphysis [Fig. 1] and the iliac crest apophysis [Fig. 2] were analyzed based on Kreitner et al. [7–9]. The process of epiphyseal fusion in the proximal humeral epiphysis [Fig. 3], distal femoral epiphysis [Fig. 4], proximal tibial epiphysis [Fig. 5], and distal tibial epiphysis [Fig. 6] was analyzed based on McKern and Steward’s classification adapted for imaging studies [10, 11]. Dental age assessment was conducted with the use of Demirjian et al. [2, 12] [Fig. 7].

Kreitner’s four-stage classification was chosen to achieve a greater clarity and ease of PMCT assessments, with stage 1 denoting no (epiphyseal/apophyseal) ossification and stage 4 denoting complete fusion [7–9]. Another classification was McKern and Steward’s classification, modified by O’Connor for imaging studies. This is a five-stage classification, with stage 0 denoting no epiphyseal fusion marked by a complete radiolucent gap between the epiphysis and diaphysis and stages 1–3 denoting the process of fusion, marked by the length of the radiolucent gap decreasing from more than half of its original length, through less than half, to its near-complete obliteration with only the metaphyseal line visible, respectively. Stage 4 denotes complete union, with all traces of the metaphyseal line gone [10, 11].

Our findings were evaluated with the use of the Digital Atlas of Skeletal Maturity by Gilsanz and Ratib, which defines the following periods of human skeletal maturity: pre-puberty (males 3–9 years old; females 2–7 years old), early and mid-puberty (males 9–14 years old, females 7–13 years old), late puberty (males 14–16 years old, females 13–15 years old), and post-puberty (males 17–19 years old, females 15–17 years old) [13]. Additionally, we evaluated our findings with respect to the age of majority, which is the legally adopted threshold of adulthood [14], with the view to showing the usefulness of postmortem examinations in forensic age estimation in the living.

A total of 234 cadavers of individuals aged from 2 to 30 years were considered for analysis in this study. These cadavers had been brought to the Forensic Medicine Department at the Jagiellonian University Medical College in the period from 2012 to 2016. Thirty-one cadavers were excluded. The exclusion criteria are as follows: any injuries involving the evaluated ossification centers and any burns that damaged bone structures. Two hundred three corpses with no bone fractures involving ossification centers were included in the analysis [Table 1]. The age range (2–30 years) was selected based on the classifications chosen for this study. Kreitner’s classification allowed the analysis of the widest age range. Additionally, the lower limit of age has been specified based on Juvenile Osteology [2] which all secondary ossification centers are recognizable. Age was determined based on identification number which containing date of birth and the known date of death.

**Table 1:** Age distribution (*n* = 203)

Chronological age [years]	Females	Males
2	2	-
3	1	2
4	2	-
5	1	1
6	1	-
7	1	2
8	2	-
9	1	1
10	-	3
11	1	1
12	2	-
13	1	2
14	1	2
15	-	2
16	3	3
17	3	4
18	5	6
19	2	16
20	3	18
21	3	8
22	2	11
23	2	8
24	2	9
25	-	16
26	2	10
27	2	4
28	-	7
29	1	3
30	1	17

## Results

The following abbreviations are from Tables 7, 8, 11 and 13: corp, corpses; DFE, distal femoral epiphysis; DTE, distal tibial epiphysis; IC, iliac crest; MCE, medial clavicular epiphysis; PHE, proximal humeral epiphysis; PTE, proximal tibial epiphysis.

### Analysis of ossification centers

The stage of a non-ossified medial clavicular epiphysis ends before the age of 18 years. The stage of complete fusion at the medial clavicular epiphysis is observed from the age of 18 years in males. The no ossification stage of the iliac crest apophysis and the beginning of ossification but without fusion with the iliac crest are no longer observed by the age of 18 years. Complete bone fusion at the iliac crest is observed from the age of 18 years in males. Beginning union at the proximal humeral epiphysis is completed before late puberty in both males and females (males 14–16 years old; females 13–15 years old). Active union at the proximal tibial epiphysis ends before the age of 18 in the male group. Recent union at the distal tibial epiphysis is observed after the age of 18 in the female group [Table 2].

**Table 2:** The process of ossification and fusion of secondary ossification centers, minimum and maximum age in years, the frequency of individuals, *min* minimum age; *max* maximum age; sex: male♂, female♀ ; *SD* standard deviation of age

Stages	Ossification centers	min–max age ± SD The percentage frequency of individuals; mean of age	Ossification centers	min–max age ± SD The percentage frequency of individuals; mean of age
1	Medial clavicular epiphysis	3–16 ± 4.08♂ *n* = 11 = 7.1%; 9.27♂ 2–13 ± 3.5♀ *n* = 15 = 31.9%; 6.6♀	Iliac crest apophysis	3–16 ± 4.6♂ *n* = 6 = 3.8%; 11♂ 2–12 ± 3.23♀ *n* = 14 = 29.8%; 6♀
2		13–20 ± 1.86♂ *n* = 18 = 11.5%; 17.8♂ 13–18 ± 2.65♀ *n* = 3 = 6.4%; 16♀		13–16 ± 1.2♂ *n* = 10 = 6.4%; 15.3♂ 13–14 ± 0.71♀ *n* = 2 = 4.3%; 13.5♀
3		17–26 ± 2.15♂ *n* = 46 = 29.5%; 21.2♂ 16–27 ± 3.38♀ *n* = 10 = 21.3%; 19.9♀		14–20 ± 1.53♂ *n* = 19 = 12.2%; 17.7♂ 16–20 ± 1.58♀ *n* = 6 = 12.7%; 17.5♀
4		18–30 ± 3.5♂ *n* = 81 = 51.9%; 25.4♂ 17–30 ± 3.54♀ *n* = 19 = 40.4%; 23.9♀		18–30 ± 3.66♂ *n* = 121 = 77.6%; 24♂ 17–30 ± 3.87♀ *n* = 25 = 53.2%; 23.5♀
0	Proximal humeral epiphysis	3–9 ± 3.1♂ *n* = 5 = 3.1%; 4.95♂ 2–9 ± 2.34♀ *n* = 12 = 25.5%; 4.75♀	Distal femoral epiphysis	3–9 ± 3.14♂ *n* = 9 = 10.6%; 5.8♂ 2–6 ± 1.77♀ *n* = 7 = 26.9%; 3.43♀
1		10–13 ± 1.41♂ *n* = 4 = 2.6%; 11♂ 8–11 ± 2.12♀ *n* = 2 = 4.3%; 9.5♀		10–14 ± 1.72♂ *n* = 6 = 7.1%; 11.8♂ 8 ± 0♀ *n* = 2 = 7.7%; 8♀
2		13–22 ± 2.34♂ *n* = 24 = 15.4%; 17.5♂ 12–18 ± 2.59♀ *n* = 6 = 12.8%; 14.8♀		15–20 ± 1.9♂ *n* = 11 = 12.9%; 17.3♂ 12–13 ± 0.05♀ *n* = 3 = 11.3%; 12.5♀
3		16–30 ± 3.81♂ *n* = 119 = 76.3%; 23.5♂ 16–30 ± 4.2♀ *n* = 27 = 57.4%; 22.22♀		17–30 ± 4.19♂ *n* = 59 = 69.4%; 23.1♂ 16–30 ± 4.16♀ *n* = 14 = 53.8%; 21.8♀
4		20–28 ± 3.79♂ *n* = 4 = 2.6%; 25.5♂ -♀		-♂ -♀
0	Proximal tibial epiphysis	3–9 ± 3♂ *n* = 8 = 9.6%; 5.5♂ 2–4 ± 0.75♀ *n* = 6 = 23.1%; 2.83♀	Distal tibial epiphysis	3–9 ± 3.1♂ *n* = 5 = 6.1%; 5.8♂ 2–8 ± 2.72♀ *n* = 7 = 28%; 4.14♀
1		7–14 ± 2.88♂ *n* = 5 = 6%; 11.4♂ 6–8 ± 1.16♀ *n* = 3 = 11.5%; 7.33♀		10–14 ± 2.16♂ *n* = 4 = 4.9%; 13♂ 4–6 ± 1.41♀ *n* = 2 = 8%; 5♀
2		11–17 ± 1.95♂ *n* = 4 = 4.8%; 15.1♂ 12–18 ± 2.63♀ *n* = 4 = 15.4%; 14.25♀		11–19 ± 2.58♂ *n* = 7 = 8.5%; 16♂ 13–18 ± 2.65♀ *n* = 3 = 12%; 15♀
3		17–30 ± 4.28♂ *n* = 66 = 79.5%; 23.2♂ 16–29 ± 4.09♀ *n* = 13 = 50%; 22♀		15–30 ± 4.41♂ *n* = 66 = 80.5%; 23.2♂ 16–29 ± 4.22♀ *n* = 12 = 48%; 22.2♀
4		-♂ -♀		-♂ 20 ± 0♀ *n* = 1 = 4%; 20♀

The stage of no fusion at the iliac crest apophysis was observed only in individuals under 18 years old. In most cases, partial fusion at the medial clavicular epiphysis was observed from the age of 18 years onwards. In males, the stage of complete fusion at the medial clavicular epiphysis and iliac crest apophysis is observed in those after the age of 18 years [Table 3]. The stage of no union at the proximal humeral epiphysis, distal epiphyses of the long bones of the lower limb, and the proximal tibial epiphysis has been observed only in individuals younger than 18 years old. Union process at the proximal tibial epiphysis has been observed only in individuals under 18 years old. In most cases, union process at the distal tibial epiphysis has been observed before the age of 18 years. Finished union at the proximal humeral epiphysis, distal femoral epiphysis, proximal and distal epiphyses of the tibia has been observed in most cases after the age of 18 years [Table 4].

**Table 3:** The number of males at various stages of fusion, stratified by their chronological age with respect to the 18-year threshold

Ossification center	Chronological age		Stage 1 and 2 (no fusion)	Stage 3 (partial fusion)	Stage 4 (complete fusion)
	< 18	18 ≥			
Medial clavicular epiphysis	< 18		21	2	0
	18 ≥		8	44	81
Iliac crest apophysis	< 18		16	5	2
	18 ≥		0	14	119

**Table 4:** The number of males at various stages of fusion, stratified by their chronological age with respect to the 18-year threshold

Ossification center	Chronological age		Stage 0 (no union)	Stage 1 and 2 (union process)	Stage 3 (finished union)
	< 18	18 ≥			
Proximal humeral epiphysis	< 18		5	14	4
	18 ≥		0	14	115
Distal femoral epiphysis	< 18		9	6	4
	18 ≥		0	11	55
Proximal tibial epiphysis	< 18		8	9	3
	18 ≥		0	0	63
Distal tibial epiphysis	< 18		5	9	5
	18 ≥		0	2	61

The stage of no fusion at the iliac crest apophysis was observed only in individuals under 18 years old. In females, complete fusion at the medial clavicular epiphysis and iliac crest apophysis has been observed in most cases after the age of 18 years [Table 5]. The stage of no union at the proximal humeral epiphysis, distal femoral epiphysis, and proximal and distal tibial epiphyses has been observed only in individuals younger than 18 years. Finished union at the proximal humeral epiphysis, distal femoral epiphyses, and proximal and distal tibial epiphyses has been observed in most cases after the age of 18 years [Table 6].

**Table 5:** The number of females at various stages of fusion, stratified by their chronological age with respect to the 18-year threshold

Ossification center	Chronological age		Stage 1 and 2 (no fusion)	Stage 3 (partial fusion)	Stage 4 (complete fusion)
	< 18	18 ≥			
Medial clavicular epiphysis	< 18		17	4	1
	18 ≥		1	6	18
Iliac crest apophysis	< 18		16	5	1
	18 ≥		0	1	24

**Table 6:** The number of females at various stages of fusion, stratified by their chronological age with respect to the 18-year threshold

Ossification center	Chronological age		Stage 0 (no union)	Stage 1 and 2 (union process)	Stage 3 (finished union)
	< 18	18 ≥			
Proximal humeral epiphysis	< 18		12	6	4
	18 ≥		0	2	23
Distal femoral epiphysis	< 18		7	5	1
	18 ≥		0	0	13
Proximal tibial epiphysis	< 18		6	3	4
	18 ≥		0	4	9
Distal tibial epiphysis	< 18		7	2	2
	18 ≥		0	3	10

Statistical analysis suggests progressive epiphyseal fusion at all secondary ossification centers with age. The Spearman’s correlation coefficient (Rho) was positive in all analyses, which indicates that epiphyseal fusion process progresses with age [Table 7].

**Table 7:** Relationship between age and epiphyseal fusion process, correlations significant at *p* < 0.001; *MCE* medial clavicular epiphysis, *IC* iliac crest, *PHE* proximal humeral epiphysis, *DFE* distal femoral epiphysis, *PTE* proximal tibial epiphysis, *DTE* distal tibial epiphysis

	MCE	IC	PHE	DFE	PTE	DTE
Rho	0.80	0.75	0.71	0.81	0.81	0.75

Analysis of data from all study subjects showed the strongest relationship between the two variables (age and epiphyseal fusion process) at distal femoral epiphysis and proximal tibial epiphysis (Rho = 0.81). This finding shows a very strong correlation, when interpreted according to Stanisz scale [Table 7].

Spearman’s Rho test was used to test for potential associations between the subject’s sex and the relationship between age and epiphyseal fusion process. The strongest relationship between age and ossification process was observed at proximal tibial epiphysis (Rho = 0.93) in females and medial clavicular epiphysis (Rho = 0.77) in males [Table 8].

**Table 8:** Associations between the subject’s sex and the relationship between age and epiphyseal fusion process; correlations significant at *p* < 0.001; *MCE* medial clavicular epiphysis, *IC* iliac crest, *PHE* proximal humeral epiphysis, *DFE* distal femoral epiphysis, *PTE* proximal tibial epiphysis, *DTE* distal tibial epiphysis

	MCE	IC	PHE	DFE	PTE	DTE
Females	0.87	0.85	0.87	0.82	0.93	0.83
Males	0.77	0.71	0.64	0.75	0.74	0.69

### Analysis of teeth

Studies show that the minimum age at the completion of permanent incisor and first molar formation is similar for males and females at the age of 9–10 and 8–9 years, respectively. The completion of canine, premolar, and second molar maturation is first observed at the age of 13 years in males. The period between the minimum age at the completion of maturation of incisors and first molars and the minimum age at the completion of maturation of canines, premolars, and second molars corresponds to the early and mid-puberty (9–14 years old) of male skeletal maturity [Table 9].

**Table 9:** Dental maturity age in years, based on stage H of Demirjian’s classification; *min* minimum age; *max* maximum age, *SD* standard deviation of age; indication of sex: male♂, female♀

Tooth number	min–max	mean; SD	Tooth number	min–max	mean; SD
*11*	10–30♂ 8–30♀	21,67; 4,75♂ 18,92; 6,66♀	*12*	10–30♂ 9–30♀	21.64; 4.77♂ 20.03; 6.12♀
*21*	10–30♂ 8–30♀	21,59; 4,78♂ 19.10; 6.67♀	*22*	10–30♂ 9–30♀	21.43; 4.69♂ 19.44; 6.41♀
*31*	9–30♂ 8–30♀	21.61; 4.78♂ 19.71; 6.38♀	*32*	10–30♂ 8–30♀	21.67; 4.79♂ 20.03; 6.12♀
*41*	9–30♂ 8–30♀	21.65; 4.77♂ 19.71; 6.38♀	*42*	10–30♂ 8–30♀	21.70; 4.80♂ 20.03; 6.12♀
*13*	13–30♂ 13–29♀	21.86; 4.64♂ 19.53; 6.35♀	*14*	13–30♂ 13–30♀	21.48; 4.65♂ 19.95; 5.97♀
*23*	13–30♂ 13–30♀	21.82; 4.64♂ 19.53; 6.35♀	*24*	13–30♂ 13–30♀	21.39; 4.7♂ 19.95; 5.97♀
*33*	13–30♂ 12–30♀	21.75; 4.80♂ 19.53; 6.35♀	*34*	13–30♂ 13–30♀	21.69; 4.79♂ 20.12; 5.99♀
*43*	13–30♂ 12–30♀	21.64; 4.76♂ 19.53; 6.35♀	*44*	13–30♂ 13–30♀	21.49; 4.74♂ 19.95; 5.97♀
*15*	13–30♂ 13–30♀	21.22; 4.72♂ 20.15; 5.77♀	*16*	9–30♂ 8–30♀	20.50; 5.84♂ 19.38; 6.65♀
*25*	13–30♂ 13–30♀	21.30; 4.79♂ 20.12; 5.99♀	*26*	9–30♂ 8–30♀	20.49; 5.64♂ 19.41; 6.64♀
*35*	13–30♂ 13–30♀	21.37; 4.64♂ 19.95; 5.97♀	*36*	9–30♂ 8–30♀	19.61; 6.29♂ 18.95; 6.53♀
*45*	13–30♂ 13–30♀	21.25; 4.47♂ 19.95; 5.97♀	*46*	9–30♂ 8–30♀	19.93; 6.57♂ 18.95; 6.42♀
*17*	13–30♂ 14–30♀	21.66; 4.68♂ 20.66; 5.6♀	*18*	19–30♂ 21–29♀	24.23; 3.73♂ 24.63; 2.97♀
*27*	13–30♂ 14–30♀	21.52; 4.6♂ 20.66; 5.6♀	*28*	19♂ 21–29♀	23.02; 3.4♂ 24.57; 3.15♀
*37*	13–30♂ 14–30♀	21.53; 4.52♂ 20.35; 5.54♀	*38*	17–30♂ 18–30♀	24.02; 3.77♂ 24.71; 3.82♀
*47*	13–30♂ 14–30♀	21.44; 4.57♂ 20.56; 5.63♀	*48*	17–30♂ 18–29♀	23.67; 3.6♂ 24.33; 3.37♀

Stage 1 of union at the proximal tibial epiphysis is observed before and after the age of 9 years. The maturity stage of incisors and first molars is observed from the age of 9 years [Tables 2 and 9]. These examples show that a combined analysis of tooth maturity and epiphyseal fusion facilitates more precise age estimation in males.

Stage F of third molar maturation in males is observed after the early and mid-puberty have been completed (9–14 years old), and stage G is observed after the late puberty (14–16 years old). The beginning of stages E, F, and G of third molar maturation occurs between the ages of 13–16 years. In combination with the absence of completely matured teeth (stage H), this time frame may be helpful in age estimation before the age of majority. Completely formed maxillary third molars are observed after the age of 18 years, and completely formed mandibular third molars are observed before the age of 18 years. The minimum age at which completely mature (stage H) both maxillary and mandibular third molars are observed is after the age of 18 years in both sexes [Table 10].

**Table 10:** Dental age, in years, based on the stages of the third molar maturation; indication of sex: male♂, female♀; *min* minimum age, *max* maximum age, *SD* standard deviation

Number of the teeth →	18		28		38		48	
Stage ↓	Min Max	Mean; SD	Min Max	Mean; SD	Min Max	Mean; SD	Min Max	Mean; SD
A	-♂ -♀	-♂ -♀	-♂ 8♀	-♂ 8; -♀	9♂ -♀	9.00; -♂ -♀	9♂ -♀	9.00; -♂ -♀
B	9–13♂ 8♀	10.67; 2.08♂ 8; -♀	9–13♂ -♀	11; 2.83♂ -♀	10♂ 8♀	10; -♂ 8; -♀	-♂ 8♀	-♂ 8; -♀
C	10–14♂ 14♀	12; 2.83♂ 14; -♀	10♂ 14♀	10; 0♂ 14; -♀	10–13♂ 12–14♀	13; -♂ 13; 1.41♀	10–14♂ -♀	11.75; 2.06♂ -♀
D	-♂ 12♀	-♂ 12; -♀	15–17♂ 12♀	16; 1.41♂ 12; -♀	-♂ 13♀	-♂ 13; -♀	17–19♂ 12–13♀	18; 1.41♂ 12.5; 0.71♀
E	13–22♂ 13–19♀	17.82; 2.44♂ 16.6; 2.3♀	13–19♂ 13–29♀	16.33; 1.87♂ 19; 5.07♀	13–19♂ 17–29♀	16.4; 2.3♂ 20.67; 4.59♀	13–19♂ 17–19♀	16.5; 2.07♂ 18; 1♀
F	15–19♂ 17–29♀	17.3; 1.58♂ 20.43; 4.43♀	14–21♂ 18–30♀	17; 2.31♂ 21.25; 5.85♀	15–22♂ 16–21♀	17.33; 2.73♂ 18.80; 2.17♀	15–20♂ 16–29♀	17; 2.1♂ 20.63; 4.03♀
G	16–29♂ 21–24♀	21.95; 3.63♂ 22.67; 1.53♀	15–29♂ 17–26♀	20.88; 4.07♂ 21.88; 3♀	16–30♂ 17–30♀	22.45; 4.36♂ 23.25; 4.65♀	16–29♂ 17–23♀	21; 3.63♂ 21; 2.71♀
H	19–30♂ 21–29♀	24.23; 3.73♂ 24.63; 2.97♀	19–29♂ 21–29♀	23.02; 3.4♂ 24.57; 3.15♀	17–30♂ 18–30♀	24.02; 3.77♂ 24.71; 3.82♀	17–30♂ 18–29♀	23.67; 3.6♂ 24.33; 3.37♀

In males, ossification of the medial clavicular epiphysis without fusion and partial fusion occurs both prior to and after the age of 18 years [Table 3]. In females, partial and complete fusion of the iliac crest apophysis is observed both before and after the age of 18 years [Table 5]. Therefore, the simultaneous presence of a fully matured maxillary and mandibular third molar [Table 10] and active union at the medial clavicular epiphysis and iliac crest apophysis may help achieve a more accurate age estimate.

## Intrarater reliability

To check whether the author’s method of measuring the extent of epiphyseal fusion at any given stage is valid, we used the quadratic weighted kappa [15], which helps assess the reliability of qualitative assessments. This coefficient can be used to evaluate the agreement of two measurements of an ordinal variable. It illustrates to what extent interrater measurements are consistent [16]. The level of interrater agreement represented by the kappa coefficient was assessed according to the interpretation presented by McHugh [17].

In this study, extent of epiphyseal fusion was assessed twice 1 year apart. Both assessments were performed by the same rater who had undergone appropriate training that allowed him to reliably assess the bones. The table below shows the agreement between the measurements performed by the same rater.

In half of the examined areas, the agreement was strong, and at the IC and DFE, it was almost perfect. The conducted analyses demonstrated that the measuring method presented in this paper is appropriate for measuring epiphyseal maturation and fusion [Table 11].

**Table 11:** Value of Kappa for ossification centers—intrarater reliability; MCE medial clavicular epiphysis, IC iliac crest, PHE proximal humeral epiphysis, DFE distal femoral epiphysis, PTE proximal tibial epiphysis, DTE distal tibial epiphysis

Ossification center	MCE	IC	PHE	DFE	PTE	DTE
Weighted kappa (κ_w_) coefficient	0.89*	0.97*	0.78*	1.00*	0.56*	0.90*
Strength of agreement	Strong	Almost perfect	Moderate	Almost perfect	Weak	Strong

*p < 0.001

The quadratic weighted kappa was also used to check whether the method of measuring tooth growth at any given stage is valid.

The kappa coefficient was calculated for the third molars [Table 12].

**Table 12:** Kappa values for third molars—intrarater reliability

Tooth numbers →	18	28	38	48
Weighted kappa (κw) coefficient	0.95*	0.99*	0.96*	0.95*
Strength of agreement	Almost perfect	Almost perfect	Almost perfect	Almost perfect

*p < 0.001

Due to the fact that the assessed parameter values remained at the same level for the remaining teeth, it was not possible to calculate the quadratic weighted kappa coefficient. Nonetheless, in light of the homogeneity of the results, the highest levels of intrarater agreement may be assumed.

These results indicate an almost perfect agreement between the measurements in all the analyzed teeth. The conducted analyses showed that the evaluated measurement method is appropriate for analyzing dental maturity.

## Discussion

Our analysis demonstrates that the stage of complete fusion in the medial clavicular epiphysis can be observed in males starting from the age of 18 years. This is consistent with the observations by other authors [18–24, 27–29]. In three studies [21, 23, 25], no ossification process is no longer observed after 18 years of age in either sex. Only in two articles [21, 28] in males fusion process started from the age of 18 years. Present study demonstrates that the no ossification process, ossification process, and finished ossification process were observed in 18 years old in either sex [Table 13].

**Table 13:** Comparison of our findings with those of other radiographic studies; indication of sex: male♂, female♀; *min* minimum, *max* maximum, *corp* corpses; MCE medial clavicular epiphysis, IC iliac crest, PHE proximal humeral epiphysis, DFE distal femoral epiphysis, PTE proximal tibial epiphysis, DTE distal tibial epiphysis

Methods, ossification centers and age groups	Authors	Male ♂ Female♀ No ossification process Max. age (years)	Male♂ Female♀ Ossification process Min.–max. age (years)		Male♂ Female♀ Finished ossification process Min. age (years)
X-ray MCE 16–30	Schmeling et al. [18] Germany	-*♂*	16.7*♂*	24.0*♂*	21.3*♂*
		-♀	16.0♀	26.8♀	20.0♀
X-ray MCE 15–30	Wittschieber et al. [19] Germany	-*♂*	16.1*♂*	29.7*♂*	22.5*♂*
		-♀	15.0♀	30.6♀	21.1♀
CT MCE 15–30	Houpert et al. [20] France	20*♂*	17.4*♂*	25*♂*	19.4*♂*
		20♀	17.2♀	24.6♀	22.3♀
CT MCE 10–25	Shedge et al. [21] India	16.10*♂*	18.71*♂*	32.88*♂*	19.94*♂*
		16.93♀	18.76♀	30.07♀	20.17♀
CT MCE 15–25,99	Zhang et al. [22] China	20.63*♂*	16.74*♂*	25.97*♂*	20.03*♂*
		20.13♀	16.28♀	25.82♀	18.89♀
CT MCE 12–30corp	Torimitsu et al. [23] Japan	17.3*♂*	16.3*♂*	25.0*♂*	19.8*♂*
		16.2♀	17.3♀	22.8♀	19.4♀
CT MCE 10–35	Ekizoglu et al. [24] Turkey	25*♂*	16*♂*	25*♂*	20*♂*
		21♀	16♀	29♀	20♀
CT MCE 13–28	Ekizoglu et al. [25] Turkey	17*♂*	17*♂*	25*♂*	-*♂*
		16♀	16♀	24♀	-♀
CT MCE 13–28	Gurses et al. [26] Turkey	21.56*♂*	17.25*♂*	27.74*♂*	-*♂*
		21.17♀	16.98♀	26.15♀	-♀
CT MCE 10–35	Kellinghaus et al. [27] Germany	20.26*♂*	17.53*♂*	26.15*♂*	21.56*♂*
		19.29♀	16.75♀	26.15♀	21.92♀
CT MCE 12.9–27 corp	Tangmose et al. [28] Denmark	24.10*♂*	18.03*♂*	25.72*♂*	21.56*♂*
		18.92♀	16.9♀	24.73♀	21.92♀
CT MCE 15–25	Bassed et al. [29] Australia	22.85*♂*	16.87*♂*	23.71*♂*	18.34*♂*
		20.01♀	16.47♀	23.91♀	18.7♀
CT MCE 2–30	Present study Poland	20*♂*	17*♂*	26*♂*	18*♂*
		18♀	16♀	27♀	17♀
X-ray IC 10–26	Fan et al. [30] China	19.0*♂*	14.38*♂*	25.28*♂*	16.70*♂*
		18.29♀	14.41♀	25.69♀	18.0♀
X-ray IC 14–26	Zhang et al. [31] China	19.0*♂*	15.31*♂*	25.84*♂*	17.95*♂*
		18.29♀	14.46♀	25.71♀	18.36♀
X-ray IC 10–25	Bartolini et al. [32] Italy	18.56*♂*	14.07*♂*	19.99*♂*	15.07*♂*
		16.78♀	12.52♀	20.45♀	15.29♀
X-ray IC 10–25 US	Bartolini et al. [32] Italy	14.86*♂*	13.64*♂*	18.56*♂*	15.07*♂*
		13.35♀	12.85♀	16.78♀	12.52♀
x-ray IC 10–30	Wittschieber et al. [33] Germany	RS 17.1*♂* LS 14.9*♂*	RS 14.3*♂* LS 15.7*♂*	RS 24.5*♂* LS 20.2*♂*	RS 17.9*♂* LS 17.9*♂*
		RS 18.5♀ LS 15.1♀	RS 13.8♀ LS 13.8♀	RS 20.6♀ LS 20.6♀	RS 16.4♀ LS 16.4♀
CT IC 10–29	Ekizoglu et al. [8] Turkey	RS 17.3*♂* LS 17.3*♂*	RS 14.5*♂* LS 14.5*♂*	RS 19.3*♂* LS 19.3*♂*	RS 17.3*♂* LS 17.3*♂*
		RS 17.9♀ LS 17.9♀	RS 14.0♀ LS 14.0♀	RS 21.6♀ LS 21.6♀	RS 17.8♀ LS 18♀
CT IC 10–29	Norouzi et al. [9] Iran	RS 26*♂* LS 26*♂*	RS 14.5*♂* LS 14.5*♂*	RS 19.3*♂* LS 19.3*♂*	RS 17*♂* LS 17*♂*
		RS 25♀ LS 25♀	RS 15♀ LS 15♀	RS 29♀ LS 29♀	RS 20♀ LS 18♀
CT IC 2–30	Present study Poland	16*♂*	15*♂*	20*♂*	18*♂*
		14♀	16♀	20♀	17♀
X-ray DFE 10–20	Aly et al. [11] Egypt	15–15.9*♂*	10–10.9*♂*	19–19.5*♂*	14–14.9*♂*
		12–12.9♀	10–10.9♀	20–20.9♀	14–14.9♀
X-ray DFE 9–19	O’Connor et al. [10] Ireland	13.7*♂*	12*♂*	18.1*♂*	14.7*♂*
		11.6♀	10.2♀	16.9♀	14.1♀
X-ray DFE 11–25	Fan et al. [34] China	12.36*♂*	11.0*♂*	16.73*♂*	14.59*♂*
		12.84♀	11.22♀	14.86♀	13.00♀
CT DFE 2–30	Present study Poland	9*♂*	10*♂*	20*♂*	17*♂*
		6♀	8♀	13♀	16♀
X-ray PTE 10–20	Aly et al. [11] Egypt	14–14.9*♂*	10–10.9*♂*	19–19.5*♂*	14–14.9*♂*
		12–12.9♀	10–10.9♀	20–20.9♀	14–14.9♀
X-ray PTE 9–19	O’Connor et al. [10] Ireland	14.5*♂*	12.0*♂*	17.8*♂*	14.7*♂*
		11.6♀	10.2♀	14.4♀	14.0♀
X-ray PTE 11–25	Fan et al. [34] China	12.99*♂*	11.47*♂*	17.07*♂*	13.03*♂*
		12.88♀	11.22♀	15.25♀	13.82♀
CT PTE 2–30	Present study Poland	9*♂*	7*♂*	17*♂*	17*♂*
		4♀	6♀	18♀	16♀

The no fusion stage of the iliac crest apophysis was not observed after 18 years old in either sex. Most of the other studies used in our comparison demonstrated similar observations in females [8, 30–33]. Moreover, in some articles [8, 30–33], the no fusion stage of the iliac crest apophysis in males was observed before the end of the post-pubertal period (17–19 years old). Norouzi reported the no fusion stage of the iliac crest apophysis in both sexes after the age of 19 years [9] [Table 13].

Ossification process at the distal femoral epiphysis in females is no longer observed from the age of 18 years [10, 34] [Table 13].

Active union at the proximal tibial epiphysis in males is no longer observed until the age of 18 years onward. This is consistent with the findings of the present study and those reported by two other authors [10, 34]. In females group active union at the proximal tibial epiphysis was observed until the age of 18 in our study and 20.9 years in another study [11] [Table 13].

Most of the authors considered in our comparison [35–37, 39] reported third molar maturity from the age of 18 years. These observations are consistent with those in the present study, except for mandibular third molar teeth in males, which had been reported to reach maturity prior to the age of 18 years [Table 14].

**Table 14:** Comparison of the findings of the present study with those of other studies in terms of third molar maturation based on Demirjian’s method of dental age estimation; sex: male♂, female♀

Authors Countries Ages	Tooth Stage Methods	Minimum age	Maximum age
Demirturk Kocasarac et al. [35] Turkey 8–25	Third molar Stage H CT	19*♂* 18♀	25*♂* 25♀
Cantekin et al. [36] Turkey 9–25	Tooth 38/48 Stage H CT	18*♂* 18.30♀	24.9*♂* 24.80♀
Bassed et al. [37] Australia 15–25	Tooth 38/48 Stage H CT	18*♂* 19♀	25*♂* 25♀
Johan et al. [38] Malaysia 14–25	Tooth 38 Stage H x-ray	18*♂* 18♀	25*♂* 25♀
Johan et al. [38] Malaysia 14–25	Tooth 48 Stage H x-ray	18*♂* 17♀	25*♂* 25♀
Streckbein et al. [39] Germany 15–22	Third molar Stage H x-ray	18*♂* 18♀	22*♂* 22♀
Present study Poland 2–30	Tooth 18/28 Stage H CT	19*♂* 21♀	30*♂* 29♀
Present study Poland 2–30	Tooth 38/48 Stage H CT	17*♂* 18♀	30*♂* 30♀

Results of our study show the importance of concurrently evaluating skeletal and dental maturation with a subsequent comparison of the findings to obtain a more accurate age estimate. Plenty of authors [29, 35, 40–42] indicate the positive value of the comparison studies between skeletal and dental age assessment. We plan in the future comparison study based on a multifactorial Bassed et al. [29] method to compare the results.

## Conclusions

This was a retrospective study in children, adolescents, and young adults. PMCT scans performed to estimate age were used to analyze the process of secondary ossification center fusion and the process of permanent tooth maturation based on published classifications [7, 10, 12]. Statistical analysis suggests progressive epiphyseal fusion at all secondary ossification centers with age. The strongest relationship between age and ossification process was observed at proximal tibial epiphysis in females and medial clavicular epiphysis in males group. A comparison of the findings obtained in the Polish population of children, adolescents, and young adults with the findings of other studies in similar age groups showed a number of similarities in terms of the time windows characterizing skeletal and dental maturation. These similarities may be helpful in age estimation [8–11, 18–39].

The limitations of our study include the relatively small sample size, smaller than those reported by other authors [18–24, 26, 27, 29–39]. Because of that, wide range of SD can be observed. Moreover, the age group under 18 years old was represented by only 45 cadavers. This small sample is due to the scarcity of individuals in this age group who undergo postmortem examinations at our Forensic Medicine Department. We hope that our further studies on this topic will allow us to expand the number of analyzed cases in this age group. In terms of the number of individuals assessed at the Forensic Medicine Department at the  Jagiellonian University Medical College, our study also revealed a disproportion in the number of evaluated males and females and only a small sample of individuals under the age of majority. These disproportions may be also due to a greater mortality among males (e.g., a greater propensity to take risks, greater proportion of suicidal attempts [43]) and the fact that fatal incidents more commonly involve adults. This situation dictates expanding similar future studies to include larger sample sizes.

The strengths of our study include the fact that other authors have typically analyzed only one ossification center or several centers in one limb or girdle in a living population [10, 11, 30, 34, 44–54]. In our study, we concurrently analyzed many ossification centers located in different parts of the skeleton. Study findings showed that a concurrent evaluation of several ossification centers yields consistent results in estimating the age. Such findings support the functionality of PMCT in corpse evaluation. Further research in cadavers is needed because the use of radiation-based imaging studies in living individuals is not recommended if it is not justified by clinical or legal indications [55, 56].

Despite the fact that the present study was conducted in corpses, the findings may be helpful not only in postmortem identification or anthropological examination but also in legal cases involving age estimation in the living. It may be used for determination of the age of illegal immigrants who do not have adequate documentation establishing their identity or estimation of the age of juvenile offenders [57].

Many authors have used MRI to assess the ossification centers in the upper and lower limbs, whereas the head and pelvis have been typically assessed via CT. This has to do with the nature of clinical practice, the types of common injuries, and the desire to use the obtained images in research studies. In this study, we used only one of these modalities (CT) to assess any given cadaver. This would not be possible in living individuals, since the extent of the scan (which included the upper and lower limbs as well as in the pectoral and pelvic girdles) would be harmful. Moreover, using a single imaging technique helped us to avoid the potential inconsistencies that would have been likely to emerge if we tried to compare the results of radiographic and non-radiographic images.

Studies also show the importance of concurrently evaluating skeletal and dental maturation with a subsequent comparison of the findings to obtain a more accurate age estimate. There are only a handful of authors who used multifactorial age estimation involving concurrent evaluation of third molars, the medial clavicular epiphysis, and the spheno-occipital synchondrosis [29, 35]. Our future research is going to focus on this method in order to compare the results. However, more research is still needed due to the high number of ossification centers.

There are a few things to keep in mind. The method chosen for the analysis of bone or radiological material should be developed on a basis similar to the one we are analyzing. It is also good how the method works on current research and the latest state of knowledge. Cunha et al. [58] noticed that, for forensic purposes, it is safer to use standardized methods, such as those described by Demirjian et al. [12], Kreitner et al. [7], Schmeling et al. [18], Kellinghaus et al. [27], and Schaefer et al. [2], which are used for body identification purposes in forensic medicine, since these methods have been shown to be effective in research studies. Moreover, the available methods should be tested on various populations and include a high number of ossification centers.

## Key points

1. This is a retrospective analysis of postmortem computed tomography images of secondary ossification centers and permanent teeth.
2. A total of 203 corpses aged 2–30 years were examined.
3. Our study showed that the data obtained for the individual ossification centers are consistent in terms of age ranges; therefore, they can be used for age estimation.
4. The results may be helpful not only in the process of postmortem identification but also in the cases involving age estimation in the living.
5. Each of the assessed secondary ossification centers shows progress in ossification stages with increasing age.
6. Combined analysis of tooth maturity and epiphyseal fusion facilitates more precise age estimation in males.
7. The minimum age at which completely mature (stage H) both maxillary and mandibular third molars are observed is after the age of 18 years in both sexes.

## Data Availability

The data presented in this study are available on request from the corresponding author.
